# Fourier Transform Infrared Spectroscopy for Typing Burkholderia cenocepacia ET12 Isolates

**DOI:** 10.1128/Spectrum.01831-21

**Published:** 2021-12-08

**Authors:** Kevin R. Barker, Michael Santino, John J. LiPuma, Elizabeth Tullis, Matthew P. Muller, Larissa M. Matukas, Manal Tadros

**Affiliations:** a Department of Laboratory Medicine and Pathobiology, University of Toronto, Toronto, Ontario, Canada; b Division of Microbiology, Department of Laboratory Medicine, Unity Health Toronto, Toronto, Ontario, Canada; c Department of Scientific Affairs, Bruker Daltonics, Billerica, Massachusetts, USA; d Department of Pediatrics, University of Michigan Medical School, Ann Arbor, Michigan, USA; e Division of Respirology, Adult Cystic Fibrosis Centre, St. Michael’s Hospital, Toronto, Ontario, Canada; f Division of Infectious Diseases, Department of Medicine, Unity Health Toronto, Toronto, Ontario, Canada; University of Arizona/Banner Health

**Keywords:** *Burkholderia*, *Burkholderia cenocepacia*, ClinProTools, ET12, FTIR, Fourier transform infrared spectroscopy, cystic fibrosis, mass spectrometry, outbreak, typing

## Abstract

The IR Biotyper and matrix-assisted laser desorption ionization–time of flight mass spectrometry (MALDI-TOF MS) using ClinProTools software (MALDI-TOF MS–ClinProTools) are two novel typing methods that rely on the analysis of carbohydrate and peptide residues in intact bacterial cells. These two methods have shown promising results in the rapid and accurate typing of bacteria. In this study, we evaluated these novel typing methods in comparison with genotypic typing for cluster analysis of Burkholderia cenocepacia epidemic strain ET12, isolated from adult cystic fibrosis patients. Sixty-six isolates of B. cenocepacia were used in this study, 35 of which were identified as the ET12 strain and 31 as non-ET12 strains by repetitive-element PCR (rep-PCR). Twelve isolates were used for the creation of typing models using IR Biotyper and MALDI-TOF MS–ClinProTools, and 54 isolates were used for external validation of the typing models. The IR Biotyper linear discriminant analysis (LDA) model had a diagnostic sensitivity of 84.6% for typing the epidemic strain, ET12. At a cutoff of 70%, MALDI-TOF MS–ClinProTools had 87.5% diagnostic sensitivity in detecting the ET12 strain (*P = *1.00). Both methods had a diagnostic specificity of ≥80% for detecting the ET12 strain. In conclusion, IR Biotyper and MALDI-TOF MS–ClinProTools offer rapid typing using proteomics and analysis of small cellular molecules with a low running cost. Our pilot study showed suboptimal accuracy of both methods for typing outbreak strains of B. cenocepacia. Extending the spectral region analyzed by the IR Biotyper can improve the accuracy and has the potential of improving the generalizability of this technique for typing other organisms.

**IMPORTANCE** Respiratory infections due to Burkholderia cenocepacia, particularly the ET12 epidemic strain, are considered sentinel events for persons with cystic fibrosis, as they are often associated with person-to-person transmission and accelerated decline in lung function and early mortality. Current typing methods are generally only available at reference centers, with long turn-around-times, which can affect the identification of outbreaks and critical patient triage. This pilot study aims to add to the growing literature illustrating the potential utility of Fourier transform infrared spectroscopy (FTIR), a novel rapid method, for the successful typing of clinically significant bacteria. In this study, we evaluated its utility to discriminate between the ET12 clone and non-ET12 isolates of B. cenocepacia and compared it to proteomics cluster analysis using MALDI-TOF MS and ClinProTools software. Both methods had encouraging but suboptimal accuracy (≥85% sensitivity and ≥83% specificity), which will likely be improved by extending the spectral region analyzed by the IR Biotyper with updated software.

## INTRODUCTION

Respiratory infections due to Burkholderia cenocepacia, particularly the ET12 epidemic strain, are considered sentinel events for persons with cystic fibrosis (CF), as they are often associated with accelerated decline in lung function and early mortality (1). Patient-to-patient transmission of this strain has been reported (1). Molecular methods like pulsed-field gel electrophoresis (PFGE), repetitive-element PCR (rep-PCR) fingerprinting, and multilocus sequence typing (MLST) are standard methods for genotyping B. cenocepacia to ascertain strain-level identification (2, 3). These methods are generally only available at reference centers, which affects turnaround time and may delay identification of outbreaks. There is growing literature on the successful use of proteomics or analyses of other molecules to assess strain-level discrimination of bacteria.

Fourier transform infrared spectroscopy (FTIR) discriminates isolates by comparing differences in carbohydrates and glycoproteins. FTIR instruments have a considerable capital cost but a low running cost and can process specimens in approximately 3 h. FTIR has had success in categorizing Pseudomonas aeruginosa, Klebsiella pneumoniae, and Enterobacter cloacae into the correct sequence types (STs) and into clusters from outbreaks (4, 5). FTIR has also been shown to successfully predict serotypes of Streptococcus pneumoniae (6). Matrix-assisted laser desorption ionization–time-of-flight mass spectrometry (MALDI-TOF MS) using ClinProTools software (MALDI-TOF MS–ClinProTools) has also shown promise with successful typing of isolates (7–10).

In this study, we evaluated the utility of FTIR using the Bruker IR Biotyper (Bruker Daltonics, Bremen, Germany) to discriminate between the ET12 clone and non-ET12 isolates of B. cenocepacia and compared it to proteomics cluster analysis using the microflex LT 60 instrument (Bruker Daltonics, Bremen, Germany) for MALDI-TOF MS–ClinProTools. Both methods were compared to molecular typing using BOX-PCR as the standard typing method. BOX-PCR is a method of typing based on analyzing the BOX dispersed-repeat motif (3).

## RESULTS

### Internal validation results.

The same isolates were used for internal validation of both IR Biotyper and MALDI-TOF MS–ClinProTools. For the IR Biotyper typing models, linear discriminant analysis (LDA), artificial neural network (ANN), and principal component analysis (PCA) had internal validation values of 77%, 85%, and 85%, respectively. One non-ET12 isolate was incorrectly typed as ET12 by all three models, one non-ET12 isolate was correctly typed as non-ET12 only by PCA, and one ET12 isolate was correct (ET12) only by ANN. The MALDI-TOF MS–ClinProTools support vector machine (SVM) typing model had an internal validation value of 95%.

### External validation results.

For the IR Biotyper, all three models (LDA, PCA, and ANN) were tested on the same isolates (*n* = 54). For the MALDI-TOF MS–ClinProTools external validation, three isolates included in the IR Biotyper external validation were not included. Three additional isolates not included in the IR Biotyper external validation were included in the MALDI-TOF MS–ClinProTools external validation. Fifty-one isolates were typed by both the IR Biotyper and MALDI-TOF MS–ClinProTools. Within the IR Biotyper results, after excluding the inconclusive results from the analysis, LDA and ANN had the highest overall accuracy of 82.7% (43/52 isolates) compared to the results for BOX PCR. LDA had higher diagnostic sensitivity (85.7%) but lower diagnostic specificity (79.2%) than ANN and PCA for detecting the ET12 strain (Table S1 in the supplemental material).

For the 54 isolates that were run using MALDI-TOF MS–ClinProTools with the SVM model, we compared a cutoff of 70% with a cutoff of 80% (a cutoff value for classification reflects the proportion of spectra for a given isolate that must be within a class to designate that isolate as belonging to that class). After excluding inconclusive results from the analysis, the overall accuracy was 87.8% (43/49 isolates) for the 70% cutoff versus 88.4% (38/43 isolates) for the 80% cutoff. For the detection of the ET12 strain, the diagnostic sensitivities were 88.9% and 90.9% for SVM cutoffs of 70% and 80%, respectively, and the diagnostic specificities were 86.4% and 85.4% for SVM cutoffs of 70% and 80%, respectively. However, SVM at the 80% cutoff had 11 inconclusive results (20.3%), compared to 5 (9.2%) for the 70% cutoff (Table S2). We next compared the performance of the models for the 51 isolates that were run on all four models after excluding inconclusive results (Table 1 and 2). The LDA model of the IR Biotyper correctly typed 41/49 isolates (83.7%), whereas MALDI-TOF MS–ClinProTools SVM at a cutoff of 70% correctly typed 40 of 46 isolates (87.0%), and at a cutoff of 80% correctly typed 35 of 40 (87.5%). The difference in accuracy between LDA and MALDI-TOF MS–ClinProTools SVM was not statistically significant (*P = *0.87). For the detection of the ET12 strain, LDA had a diagnostic sensitivity of 84.6%, while SVM had diagnostic sensitivities of 87.5% and 89.5% at cutoffs of 70% and 80%, respectively. These differences were not statistically significant (*P = *0.88). The diagnostic specificities were 82.6% for LDA and 86.4% and 85.7% for SVM at cutoffs of 70% and 80%, respectively (Table 3). These differences were not statistically significant (*P = *1.00).

**TABLE 1:** Accuracy of the IR Biotyper and MALDI-TOF MS with various typing models for the identification of B. cenocepacia ET12 and non-ET12 isolates

Class(es) (no. of isolates)^*a*^	No. (%) of isolates identified by:				
	IR Biotyper with^*b*^:			MALDI-TOF MS–ClinProTools with SVM at a cutoff of^*c*^:	
	LDA	ANN	PCA	80%	70%
Class 1, non-ET12 (*n* = 24)	19 (82.6)	20 (87.0)	21 (91.3)	18 (85.7)	19 (86.4)
Class 2, ET12 (*n* = 27)	22 (84.6)	21 (80.8)	16 (64.0)	17 (89.5)	21 (87.5)
Class 1 + 2 (*n* = 51)	41 (83.7)	41 (83.7)	37 (77.1)	35 (87.5)	40 (87.0)

a Only isolates used for all methods were included in the external validation set (*n* = 51), with 3 inconclusive isolates excluded.

b LDA, linear discriminant analysis; ANN, artificial neural network; PCA, principal-component analysis.

c SVM, support vector machine.

**TABLE 2:** Numbers of ET12 (*n* = 27) isolates with concordant, discordant, and inconclusive identification by the IR Biotyper and MALDI-TOF MS with various typing models

Classification (*n* = 27 isolates)^*a*^	ANN class call color code	No. (%) of isolates identified by:				
		IR Biotyper with^*b*^:			MALDI-TOF MS–ClinProTools with SVM at a cutoff of^*c*^:	
		LDA	ANN	PCA	80%	70%
Concordant	Green	22 (81.5)	21 (77.8)	16 (59.3)	17 (63.0)	21 (77.8)
Discordant	Red	4 (14.8)	5 (18.5)	9 (37.5)	2 (7.4)	3 (11.1)
Inconclusive	Yellow	1 (3.7)	1 (3.7)	2 (7.4)	8 (29.6)	3 (11.1)

a Only isolates used for all methods were included in the external validation set.

b LDA, linear discriminant analysis; ANN, artificial neural network; PCA, principal-component analysis.

c SVM, support vector machine.

**TABLE 3:** Performance characteristics of the IR Biotyper and MALDI-TOF MS with various typing models for discrimination between BOX PCR-confirmed ET12 and non-ET12 strains of Burkholderia cenocepacia

Isolates (*n* = 51)^*a*^	Value (%) using:				
	IR Biotyper with^*b*^:			MALDI-TOF MS–ClinProTools with SVM at a cutoff of^*c*^:	
	LDA	ANN	PCA	80%	70%
Diagnostic sensitivity	84.6	80.8	64.0	89.5	87.5
Diagnostic specificity	82.6	87.0	91.3	85.7	86.4
Positive predictive value	84.6	87.5	88.9	85.0	87.5
Negative predictive value	82.6	80.0	70.0	90.0	86.4

a Only isolates used for all methods were included in the external validation set, with 3 inconclusive isolates excluded.

b LDA, linear discriminant analysis; ANN, artificial neural network; PCA, principal-component analysis.

c SVM, support vector machine.

## DISCUSSION

Early identification of patients harboring B. cenocepacia ET12 is crucial, as they are more likely to have acquired this organism by person-to-person transmission, which requires robust contact tracing, cohorting, and other infection control practices. Additionally, these patients are more likely to require aggressive antibiotic therapy. A number of genotyping methods have been developed during the past few decades, including ribotyping (11), PFGE (12), rep-PCR (13), and MLST (multilocus sequence typing) (14). More recently, multilocus variable number repeat analysis (MLVA) and whole-genome sequencing have been utilized for genotyping (1, 15). In this study, we looked to investigate two novel, rapid methods of typing Burkholderia cenocepacia into two groups: the epidemic ET12 strain and non-ET12 strains. We used MALDI-TOF MS–ClinProTools using the SVM model and the IR Biotyper using the LDA, ANN, and PCA models. The IR Biotyper technology has been applied in typing S. pneumoniae, Gram-negative bacilli during hospital outbreaks, methicillin-resistant Staphylococcus aureus (MRSA), and others (6, 16, 17).

Our study is a proof-of-concept study that will need further prospective verification. It showed equivalent performance of the IR Biotyper and MALDI-TOF MS–ClinProTools in detecting the epidemic strain of B. cenocepacia, ET12. Identification of an isolate as presumptively ET12 by IR Biotyper or MALDI-TOF MS–ClinProTools would trigger infection control interventions and assist in halting hospital transmission of this serious clone while waiting for molecular confirmation.

The LDA model seemed to be the typing tool with the best results within the IR Biotyper results. However, its performance was still suboptimal. This could be because the data were collected using an earlier version of the software which truncates the spectra analyzed to the 1,300 to 800 cm^−1^ range, allowing only the carbohydrate portion of the isolate to be analyzed. A newer version of the software allows the selection of three wave regions of interest per analysis, using the splicing method. This capability might, in theory, improve the accuracy of the IR Biotyper. Although LDA had the fewest discordant identifications for ET12 isolates among the methodologies used with the IR Biotyper, MALDI-TOF MS–ClinProTools using the SVM model with a cutoff of 80% had the fewest discordant identifications for ET12 isolates overall but the highest number of inconclusive results (*n* = 8).

Discrepant identifications for ET12 and non-ET12 isolates were investigated further. One isolate, a non-ET12 strain, was identified as ET12 by all three IR Biotyper models, but its identification was inconclusive by MALDI-TOF MS–ClinProTools with the SVM model.

For construction of both the LDA and ANN classifiers, the ET12 and non-ET12 training data set principal components were used as the inputs. LDA strives to find a new axis through the principal components that most efficiently maximizes the separation between the class mean values while also decreasing within-class variance. Once this new discriminator*y* axis is found, the principal components are projected onto it. We hypothesize that this intrinsic attribute, decreased within-class variance, is what made LDA the most successful model overall for this particular problem.

**FIG 1:**
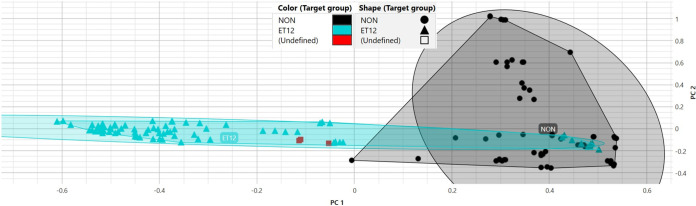
Principle component analysis (PCA) model illustrating class 1 (non-ET12) and class 2 (ET12) isolates and unknown isolates’ replicates. LDA class 1 isolates are illustrated as black circles (NON), class 2 isolates as cyan triangles (ET12), and unknown isolates’ replicates (*n* = 3) as red squares (Undefined). Unknown isolates’ replicates are clustered within class 2 (ET12). Convex hulls illustrate polygons enclosing all data points of the group, while ellipses illustrate the average position of the group. Each spectrum is plotted according to its PC1 versus PC2 (principal component) values for *x* versus *y*, respectively.

**FIG 2:**
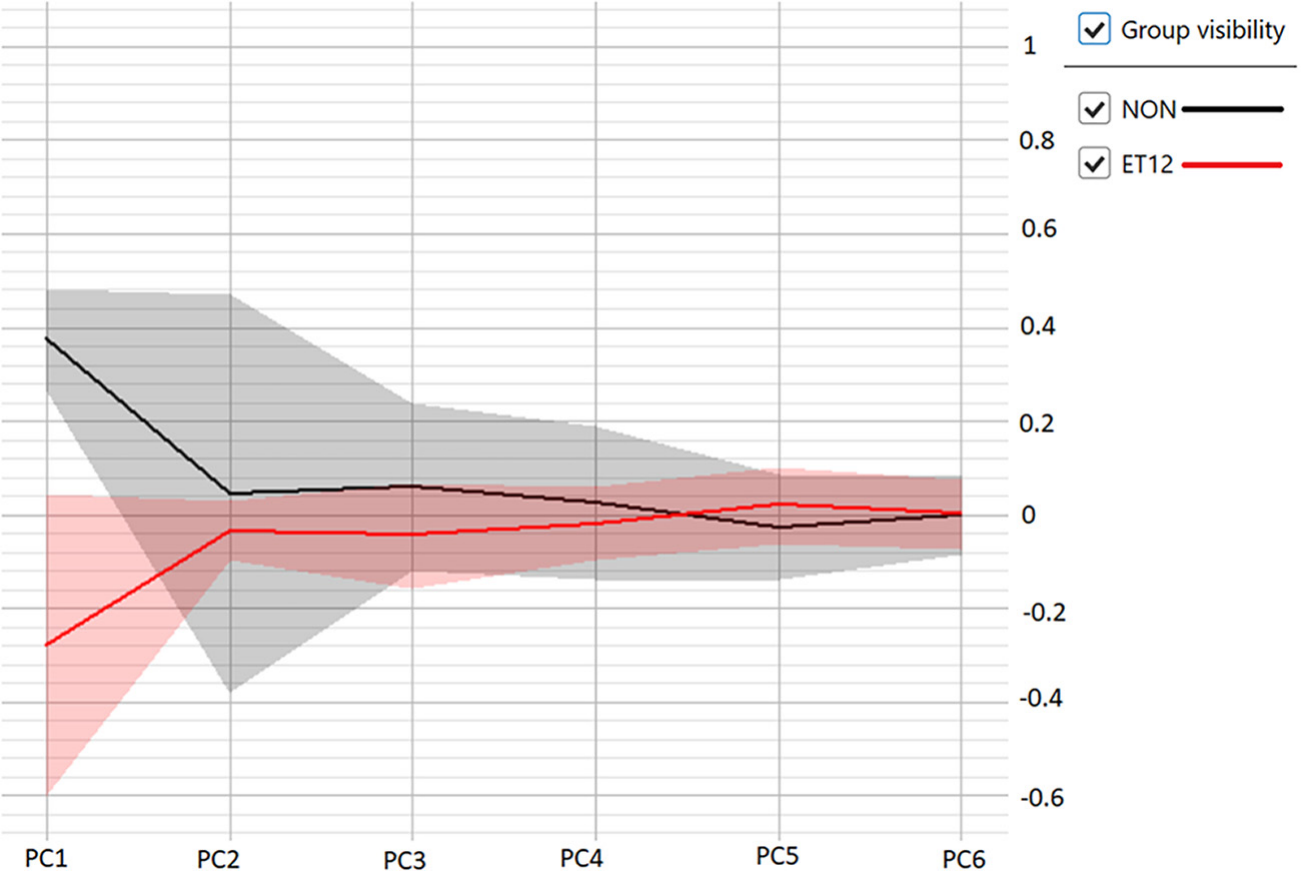
Deviation plot to determine of the number of principle components to be used for the artificial neural network (ANN) classifier. The *x* axis represents the principal components used to differentiate between the two classes, while the *y* axis represents the level of variation contained within each component. The solid lines represent the average value for each component, while the shaded areas indicate the standard deviation of each component. Class 1 is illustrated by the black line and shaded area (NON), while class 2 is illustrated by the red line and shaded area (ET12).

One isolate in the ET12 training set, isolate 24, behaved spectrally much more like the non-ET12 isolates. This can be visualized on the PCA scatterplot shown in Fig. 1. Isolate 24 can be seen at approximately 0.4 along principal component 1 (PC1), or the *x* axis, while most measurements for the ET12 training set are located at approximately −0.4, quite a distance away. The LDA was likely better able to compensate for this within-class variance than the ANN. The deviation can also be visualized within the deviation plot shown in Fig. 2. We can see that for the ET12 class, there is a large standard deviation within the first principal component, represented by the area shaded in red. Considering that the first principal component contains most of the variation contained within the data set, this was likely a source of struggle for the ANN classifier when predicting whether an unknown belonged to the ET12 class. With a training set of six isolates per class, a significant outlier like isolate 24 has a large impact on data spread and hinders a classifier’s ability to predict unknowns. In machine learning applications, it is commonly recommended to use a minimum of 10 isolates, or examples, per class for this reason (18). The relatively small size of the training sets for the typing models is considered one limitation of our study. Other limitations included retrospective typing of archived isolates using IR Biotyper and MALDI-TOF MS–ClinProTools, whereas the “gold standard” typing method was undertaken prospectively. CF patients have been shown to be colonized by Pseudomonas aeruginosa strains with diverse phenotypic heterogeneity (19). Many of our study patients were colonized simultaneously with multiple morphotypes of B. cenocepacia and, potentially, both ET12 and non-ET12 isolates. Therefore, it is possible that the frozen isolate tested retrospectively could be a different lineage from the isolate sent prospectively for BOX PCR, leading to discrepancies. The recommended number of spectra for each isolate in each class is at least six. For some of our isolates, the number of spectra represented in the training set were limited. This was due to technical difficulties with some isolates that would not homogenize readily, leading to invalid spectra in some replicates.

Overall, the IR Biotyper and MALDI-TOF MS–ClinProTools are novel, rapid methods for the identification of ET12 lineage isolates. Flagging an isolate as a potential ET12 within hours by these methods allows quick implementation of IPAC that can mitigate the spread of this potentially fatal strain until confirmation by molecular typing is available. A high positive predictive value would therefore be advantageous in this patient cohort. The positive predictive value in our data set was 87.5% for MALDI-TOF MS–ClinProTools using the SVM model at a cutoff of 70%, and it was 84.6% for the IR Biotyper using LDA. Extending the spectral region analyzed by the IR Biotyper can have the potential of improving its accuracy, as well as the generalizability of this technique for typing other organisms, such as Pseudomonas aeruginosa.

## MATERIALS AND METHODS

### Isolates.

Sixty-six B. cenocepacia isolates were used in this study. The majority of the isolates (*n* = 48) were clinical isolates from respiratory specimens from CF patients seen at the Toronto CF Adult Center. Eighteen isolates were provided from the Burkholderia cepacia Research Laboratory and Repository at the University of Michigan. Species identification was confirmed by *recA* sequence analysis. *recA* gene amplification followed by sequencing is considered a reference method for identification of members of the Burkholderia cepacia complex to the species level (20). Genotyping for strain-level identification was performed with rep-PCR employing a BOX-A1R primer as previously described (3). Thirty-five isolates were identified as strain ET12, and 31 isolates as non-ET12 strains. Twelve isolates were used for IR Biotyper and MALDI-TOF MS–ClinProTools model creation. Fifty-four isolates were used for external validation.

### IR Biotyper.

The IR Biotyper (Bruker Daltonics, Bremen, Germany) is an FTIR system used for analyzing the vibrations of carbohydrate constituents present in many molecules, including glycoproteins. This FTIR spectrum is like a fingerprint, allowing the identification of microorganisms to the subspecies level. The system is composed of a high-performance FTIR spectrometer, a reader for 96-well silicon microplates, and OPUS software for system control and automatic measurement of spectra, as well as the IR Biotyper software, which has been specifically designed to facilitate microorganism typing using FTIR spectra.

### Preparation of IRTS.

Lyophilized infrared test standard 1 (IRTS 1) and IRTS 2 were resuspended according to the manufacturer’s recommendations. Twelve microliters each of IRTS 1 and IRTS 2 was spotted twice on the target plate with each run.

### Preparation of isolates and analysis by IR Biotyper.

Each isolate was subcultured from storage in a −70°C freezer once on blood agar. This was followed by a second subculture on tryptic soy agar and incubation for exactly 24 h in ambient air before sample preparation. Fifty microliters of 70% (vol/vol) ethanol was added to a 1.5-ml Bruker suspension vial containing metal beads (1- by 3-mm cylindrical stainless steel beads, four per reaction tube). Using a 1-μl disposable inoculation loop, an overload of the organism was taken from the confluent-growth area of the agar. The biomass was submerged into the ethanol in the suspension vial and then dislodged from the loop by flipping. The vial was then closed and vortexed for 30 to 60 s for a homogenous suspension. Fifty microliters of deionized water was then added to the vial to increase the surface tension of the suspension. After vortexing again, 15 μl of the homogenous bacterial suspension was placed on a silicon sample plate (Bruker Daltonics, Bremen, Germany). Each isolate was analyzed in five replicates. The sample plate was dried at 37°C for about 15 to 20 min. The measurements were carried out using an IR Biotyper System (Bruker Daltonics, Bremen, Germany) running the IR Biotyper software (version 3.0) with the default analysis setting to acquire a wavenumber range of 4,000 to 500 cm^−1^, which meant that 3,629 data points were acquired. The IR Biotyper software truncates these spectra to a wavenumber range of 1,300 to 800 cm^−1^. While the sample spectra are acquired, they are automatically imported into the IR Biotyper system, and a technical quality test is performed. The following parameters are determined: “absorption,” which should range from 0.4 to 2 to ensure that the detector can still measure in a linear way, and “noise,” “water vapor,” and “Fringes,” which are interference artifacts that might occur (e.g., by back-reflection of the IR light). The signals in the ranges R2 (1,700 cm^−1^ to 1,600 cm^−1^) and R3 (1,200 cm^−1^ to 960 cm^−1^) are divided by the noise and water vapor values to get the “signal/noise” and “signal/water” ratios, which should be >200 and >40, respectively, for the analysis to be acceptable. The software additionally performs a nearest-neighbor matching during the run, during which the analyzed spectra of the isolate are compared to other spectra that are already available in the system and belong to the same species.

### (i) LDA model creation.

**FIG 3:**
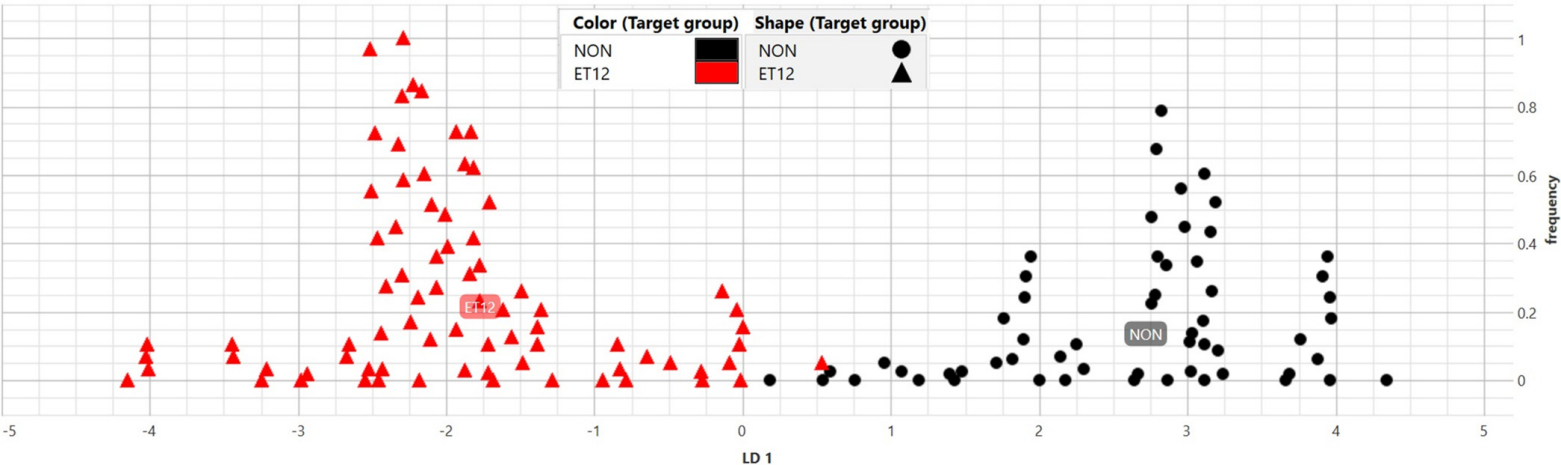
Linear discriminant analysis (LDA) model comparison of LDA class 1 (non-ET12) and LDA class 2 (ET12) isolates. LDA class 1 data points are illustrated as black circles (NON), while LDA class 2 isolates are illustrated as red triangles (ET12). The horizontal *x* axis is discriminatory between class 1 (>0) and class 2 (<0). The vertical *y* axis indicates a measure of local frequency of the data points.

LDA was used for model creation. LDA is a tool available in IR Biotyper software and has the advantage of minimizing the variance within a group and maximizing the variance between groups. For example, if variation exists between spectra of the same isolate, then LDA is a useful method to reduce noise, i.e., random differences between measurements. Six B. cenocepacia isolates of sequence types other than that of ET12, each run five times, were used to create LDA class 1. LDA class 2 was created using six ET12 isolates (Fig. 3).

### (ii) ANN model creation.

ANNs are models based on the structure and function of biological neural networks. Decisions are made by passing information between layers of nodes, or neurons, with each layer acting as both an input and an output, which allows the network to detect associations hidden within complex data sets. In the case of the IR Biotyper, the first layer of nodes is based on the principal components of the training data set. Most of the variance contained within a data set is captured within the first several principal components; however, useful discriminatory information can be found in later components. The IR Biotyper allows the use of up to 20 principal components as neural inputs. Six B. cenocepacia isolates of sequence types other than ET12, each run five times, were designated class 1. Class 2 was created using six ET12 isolates. For this data set, it was determined that the first six principal components contained the bulk of discriminatory information by viewing the deviation plot seen in Fig. 2. The model is trained through an iterative process of classifications performed by holding out training spectra and attempting to classify them. Specific neural paths are strengthened when the model arrives at a correct decision; conversely, misclassifications weaken the likelihood that the neural path which resulted in the misclassification will be followed again. During the training process, the ANN classifier completes an internal validation procedure where 75% of the training data are used to train a test classifier while the remaining 25% are held out and used to verify the classifier’s predictive ability. This process is performed four times in total so that each data point is used in the validation only once. A confusion matrix is the output result of this cross-validation study (Table S2). The final version of the classifier incorporates 100% of the training data into the prediction model. The number of training iterations is selected by the user. For this model, 400 iterations were chosen.

### Unsupervised cluster analysis using PCA.

PCA is an unsupervised data dimensionality reduction technique. The technique strives to transform a large set of variables into a smaller one while still preserving differentiating characteristics of the data set. Within the IR Biotyper, each spectrum is plotted as a point within a 2- or 3-dimensional space, depending on the user’s preference. The spectrum’s location is plotted using the values of its 1st, 2nd, and 3rd principal components as values for *x*, *y*, and *z*, respectively. Six B. cenocepacia isolates of sequence types other than ET12, each run five times, were used to create PCA class 1. PCA class 2 was created using six ET12 isolates.

### Internal validation of IR Biotyper models.

For the internal validation, we chose the method of leaving one isolate out rather than the classic method of partitioning the data into randomized subsets for training and classification (21). With this method, all but one isolate’s spectra were used to build the classification model and the omitted isolate was then classified using the resultant model. The rationale for this choice was that each isolate was represented by up to nine replicate spectra. If the data were randomly partitioned, spectra from a single isolate might end up in both the training set and the classification set, which could potentially cause bias in the results. The “leave one isolate out” method was performed for 12 iterations in total for each model.

### External validation of IR Biotyper model.

Fifty-four B. cenocepacia isolates characterized by BOX PCR as ET12 or non-ET12 were used for external validation of all three models, LDA, ANN, and PCA. These isolates were different from the ones used in model generation. Each isolate was run in three to six replicates over two to 3 days. For each run, the instrument would analyze the spectra of IRTS 1 and IRTS 2 and compare spectra of the same standard. If the analysis of the standards showed unsatisfactory results, the run would be aborted. If the analysis of standards met designated criteria, the instrument would then analyze the isolates. The accepted spectra for each isolate used for external validation would be designated unknowns and tested against classes 1 and 2. Any result greater than 3 standard deviations from the mean of the class training data was considered not classifiable.

### External validation using LDA.

**FIG 4:**
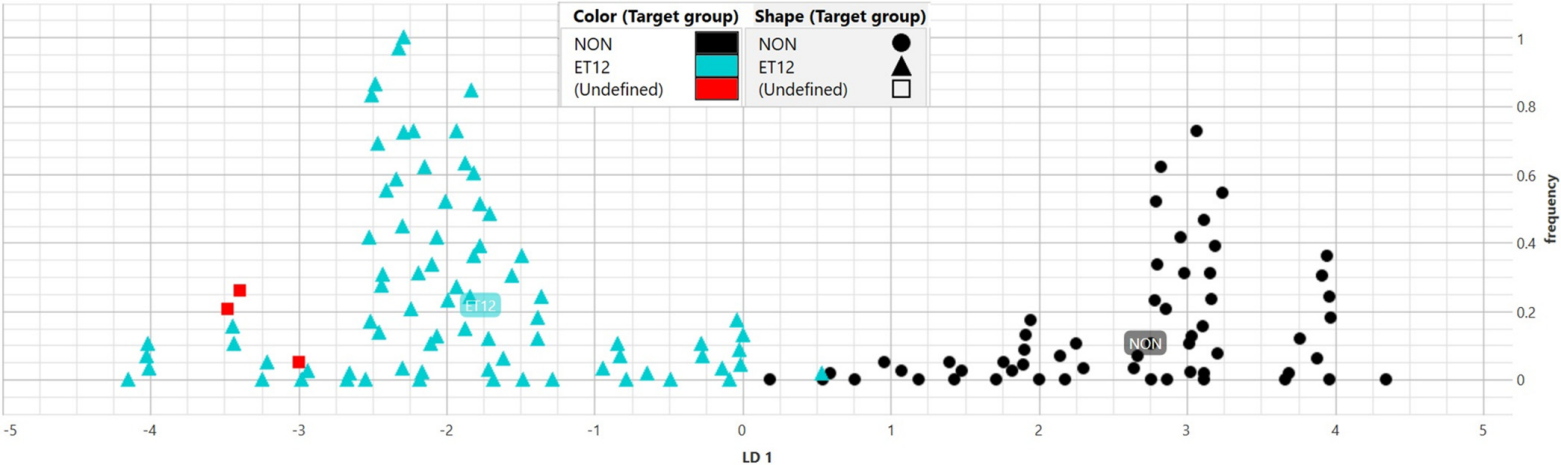
Linear discriminant analysis (LDA) model with class 1 (non-ET12), class 2 (ET12), and unknown isolates’ replicates. LDA class 1 isolates are illustrated as black circles (NON), class 2 isolates as cyan triangles (ET12), and unknown isolates’ replicates (*n* = 3) as red squares (Undefined). Unknown isolates are clustered within class 2 (ET12). The *x* axis is discriminatory between class 1 (>0) and class 2 (<0). The *y* axis indicates a measure of local frequency of the data points.

In a two-class LDA, the horizontal *x* axis discriminates class 1 (>0) from class 2 (<0), while the vertical *y* axis indicates a measure of the frequency of data points (Fig. 4).

### External validation using ANN.

Running an unknown isolate spectra against the ANN classification model results in a class call that is color coded to denote the confidence in the call. A green color-coded result indicates that the unknown spectrum is located within the spectral space of the training set, and therefore, the result can be considered reliable. A yellow color-coded result is located at the periphery of the spectral space of the training set, and therefore, the result must be viewed with caution. A red color-coded result is to be regarded as unreliable because the unknown spectra are far from what was trained. Since multiple spectra per unknown isolate were used, there were incidences where replicate spectra were classified differently. In this event, a tie breaker took place. If one class was called with a higher confidence (color) than the other, the higher-confidence class was favored. If both classes were called with equal confidence, the class with the higher number of calls was favored.

### External validation using PCA.

In PCA, the typing result is presented as a scatterplot figure with convex hulls that are polygons enclosing all data points of a group and ellipses that are centered on the average position of a group. The two radii of the ellipse are calculated assuming a bivariate normal distribution of the group’s data points. If an unknown isolate data points fell cleanly within a class’s ellipse, they were classified as such. If the unknown isolate’s data points fell outside both class’s ellipses or fell within the intersection of the ellipses, they were deemed indeterminate.

### Preparation of isolates for analysis by MALDI-TOF MS–ClinProTools.

Each isolate was taken from storage at −80°C, subcultured twice on blood agar, and incubated for 24 h in ambient air. A few colonies were picked from the blood agar plate and subjected to the tube extraction method using acid as described in CLSI M58, 1st edition (22). One microliter of the supernatant was spotted on a polished steel target plate and overlaid with 1 µl of α-cyano-4-hydroxycinnamic acid (HCCA) matrix. Each isolate was spotted 12 times.

### Creation of MALDI-TOF MS–ClinProTools typing model.

MALDI-TOF MS using a microflex LT 60 instrument (Bruker Daltonics GmbH, Bremen, Germany) and ClinProTools software version 3.0 (Bruker Daltonics) was used to generate a model for cluster analysis of the B. cenocepacia isolates. Within ClinProTools, the support vector machine (SVM) algorithm was chosen for B. cenocepacia classification based on several iterations of model creation comparing SVM to other algorithms in ClinProTools. The SVM is an artificial intelligence tool that analyses a variety of peaks and ranks them based on their separation properties. It then uses those selected peaks to generate pattern recognition for each “class” or cluster. This pattern recognition enables SVM to predict whether a new spectrum from a test isolate is related to one of the classes within the model.

The 12 isolates used for creating the IR Biotyper model were used for MALDI-TOF MS–ClinProTools model creation. Spectra generated by MALDI-TOF MS were “cleaned up” by ClinProTools using spectrum recalibration (1,000 ppm maximal peak shift, 30% match to calibrant peaks, exclusion of spectra that could not be recalibrated), average spectrum calculation (resolution, 800), average peak list calculation (signal-to-noise threshold, 5), and peak calculation in the individual spectra. The software then determined whether there were sufficient differences between classes based on differences in spectra. The typing model included two classes; class 1 was non-ET12 and class 2 was ET12. Twelve spectra were generated for each isolate.

### Internal validation of typing model using MALDI-TOF MS–ClinProTools.

Internal validation was done by dividing the data set into *k* equal parts using the cross-validation function of ClinProTools. From each data set, 20% of the data were removed and the remaining 80% were used to develop a typing model. Each model was then tested on the 20% of data not used in model development. This entire process was repeated for 10 iterations.

### External validation of typing model using MALDI-TOF MS–ClinProTools.

Fifty-four B. cenocepacia isolates not included in model creation were used to validate the typing model. Isolates for external validation were prepared using the same tube extraction method, and each was spotted 12 times on the target plate to generate 12 spectra for each isolate. External validation was performed using the validate function of ClinProTools, providing a probability for assigning the tested isolate to each class. The percentages of agreement between the results for ClinProTools and genotypic typing by BOX PCR were calculated at cutoffs of 80% and 70%. A cutoff value for classification reflects the proportion of spectra for a given isolate that must be within a class to designate that isolate as belonging to that class. A cutoff of 80% for example, means that for an isolate to belong to a class, more than 9/12 of its spectra were grouped within this particular class.

### BOX PCR.

DNA from each isolate was prepared by heating one colony at 95°C for 15 min in 20 μl of lysis buffer containing 0.25% (wt/vol) sodium dodecyl sulfate (SDS) and 0.05 M NaOH. Following lysis, 180 μl of distilled water was added, and the DNA solutions were stored at 4°C. Rep-PCR typing with a BOX-A1R primer (5′-CTACGGCAAGGCGACGCTGACG-3′) was performed as previously described (3). PCR products were separated on 25-cm-long 1.5% agarose gels in 0.5× Tris-borate-EDTA (TBE) buffer (60 mA for 4 h at room temperature). A 1-kb molecular-weight marker (Gibco, Thermo Fisher Scientific, MA, USA) was used multiple times on each gel to allow normalization. Following staining with ethidium bromide and visualization by UV illumination, gels were analyzed.

### Statistical analysis.

Pearson’s chi-square test was performed to assess differences between the accuracy, diagnostic sensitivity, and diagnostic specificity of IR Biotyper and MALDI-TOF MS–ClinProTools using R (R Core Team, 2021).
