# Supplementary material for: Fourier Transform Infrared Spectroscopy for Typing Burkholderia cenocepacia ET12 Isolates
Source: Microbiol Spectr. 2021 Dec 8;9(3):e01831-21. doi: 10.1128/Spectrum.01831-21 (PMC8653821; doi:10.1128/Spectrum.01831-21)
Supplement: SUPPLEMENTAL FILE 1 — Supplemental material. Download SPECTRUM01831-21_Supp_1_seq8.pdf, PDF file, 0.6 MB [file spectrum01831-21_supp_1_seq8.pdf]

Supplemental Material:

Table S1: Number of concordant, discordant and inconclusive identifications of *B. cenocepacia* ET12 and non-ET12 by IR Biotyper linear discriminant analysis (LDA), artificial neural network (ANN) and principle component analysis (PCA) models (n=54).

		**IR Biotyper**		
**Class (n)**		**LDA (%)**	**ANN (%)**	**PCA (%)**
Class 1: non-ET12 (n=25)	Concordant	19 (76.0)	20 (80.0)	21 (84.0)
	Discordant	5 (20.0)	4 (16.0)	3 (12.0)
	Inconclusive	1 (0.0)	1 (4.0)	1 (4.0)
Class 2: ET12 (n=29)	Concordant	24 (82.8)	23 (79.3)	18 (62.1)
	Discordant	4 (13.8)	5 (17.2)	9 (31.0)
	Inconclusive	1 (3.5)	1 (3.5)	2 (6.9)
Class 1 + 2 (n=54)	Concordant	43 (79.6)	43 (79.6)	39 (72.2)
	Discordant	9 (16.7)	9 (16.7)	12 (22.2)
	Inconclusive	2 (3.7)	2 (3.7)	3 (5.6)

- confidential -

Table S2: Number of concordant, discordant and inconclusive identifications of *B. cenocepacia* ET12 and non-ET12 by MALDI-TOF MS support vector machine (SVM) model at 80% and 70% cut-offs (n=54).

		**MALDI-TOF MS ClinProTools**	
**Class (n)**		**SVM Cut-off 80% (%)**	**SVM Cut-off 70% (%)**
Class 1: non-ET12 (n=24)	Concordant	18 (75.0)	19 (79.2)
	Discordant	3 (12.5)	3 (12.5)
	Inconclusive	3 (12.5)	2 (8.3)
Class 2: ET12 (n=30)	Concordant	20 (66.7)	24 (80.0)
	Discordant	2 (6.7)	3 (10.0)
	Inconclusive	8 (26.7)	3 (10.0)
Class 1 + 2 (n=54)	Concordant	38 (70.4)	43 (79.6)
	Discordant	5 (9.3)	6 (11.1)
	Inconclusive	11 (20.4)	5 (9.3)

- confidential -

Predicted class

Actual class	ET12	NON	Class recall
ET12	77	-	100%
NON	-	57	100%
Class precision	100%	100%	

Figure S1: Confusion matrix principle component-artificial neural network (PC-ANN) algorithm. In total the ANN training set contained 134 spectra, 77 from the ET12 class and 57 from the non-ET12 class. During training, the model was able to accurately predict the classes which hold out spectra belonged to 100% of the time for both classes. “NON” = class 1 (non-ET12); “ET12” = class 2 (ET12).
